# The Syndemic of Inequity and COVID-19 in Virtual Care

**DOI:** 10.2196/37717

**Published:** 2022-06-01

**Authors:** Esha Ray Chaudhuri

**Affiliations:** 1 Calgary, AB Canada; 2 See Acknowledgments

**Keywords:** COVID-19, virtual care, health equity, syndemic, aging, ethnicity, older adults, equity, digital health, diversity, ethnic minority groups, minority groups, older ethnic adults

## Abstract

The critical intersections of structural inequities and vulnerabilities of marginalized populations, particularly those engaging the social gradient of minority ethnic communities, are revealed in the syndemic approach to COVID-19. Although proposals for cultural interventions to improve virtual care provide relevant measures, they may not address the root cause of the disparate impacts of a pandemic on population subgroups. The common misperception of equality as synonymous with equity further impedes the efficacy of digital health in quality-of-care initiatives, as it systemically fails to acknowledge the disparate realities of marginalized populations, while intending to benefit all. This commentary suggests that an alignment of the health care system with Canada’s pluralist principles would support a paradigm shift in transforming virtual care into an equitable standard as envisioned by Pham and colleagues in their paper, “The Future of Virtual Care for Older Ethnic Adults Beyond the COVID-19 Pandemic.”

## Introduction

In a fundamental sense, the vision for transforming virtual care from that of an exclusive service that benefits only a few to that of a standard for providing equitable care for all [1] echoes the age-old debate between policy variations on the *zip code* and the *genetic code.* This commentary aims to further develop the key theme discussed by Pham et al [1]—engaging the “reimagining” of virtual care for older ethnic adults—by considering the syndemic nature of COVID-19 and the intersection of cultural interventions in care and equity in virtual care.

## The Context of the COVID-19 Syndemic

Well before the onset of COVID-19 and other major pandemics in the past 2 decades, studies on the design and evaluation of eHealth interventions recognized the challenges and implications of the interdisciplinary nature of the field [2].

The emergency management of health care in the pandemic era inadvertently proved the critical role of the social determinants of health through data about the rapid viral spread in largely marginalized, resource-challenged communities [3]. Based on the presence of similar contexts like the digital divide along pathways of structural inequities, COVID-19 has been characterized as a syndemic as opposed to a pandemic [4], emphasizing the intersections of the contributing demographic, social, economic, and environmental factors of the pandemic.

A summary review of the literature suggests that despite a significant number of health care and digital health reform initiatives, which address the disparities experienced by marginalized ethnic communities [5], few have addressed the need for a systemic transformation based on equity. Even the unique perspectives of reorienting the health care culture to its original *benevolent* foundation appear to sustain in principle the context of ethnic minority populations’ vulnerabilities [6].

Syndemic theory advances the examination of health and health care disparities while emphasizing the contexts of social and economic systems in these processes. As such, the theory provides a critical alternative to conventional systemic reform culture. It recognizes how disparities in social realities are accountable for not only *shaping* the marginal experience but also for its *distribution* across subgroups of populations.

## Aging, Ethnicity, and the Equity Paradox

In the current design, implementation, and evaluation of virtual care, the ethnicity context represents one of the several dimensions of equity, such as aging, gender, etc. To evaluate the impact of equity in digital health, it is essential that the determinants be addressed within the synergistic lens of intersectionality; the interface of the factors with structural inequities of exclusive policies presents an additional dimension.

With the increasing diversity of the population, the need to recognize equity becomes imperative. The perception of ethnic diversity as a “strength” and “asset” as found in different sectors of social planning, such as business, industry, or labor, provides an important contrast to the typical safety-net approaches to vulnerable populations of ethnic minorities in health equity and digital health studies [7].

Within studies about the intersections of ethnicity, aging, and equity [5], inclusion of ethnic older adults enhances the generic minority data measured by metrics that assume homogeneity of vulnerabilities. The ethnically nuanced care expectations of older adults and, more importantly, the cultural values that frame the expectations, are seldom contextually related to any specific equity dimension.

Pham et al [1] offer a good example of leveraging the strength of ethnic diversity to enhance quality of care. The paper presents important insights about unique cultural elements of filial piety and kinship values prevalent in Asian families. The distinct cultural norms explain the common practice adopted by family members, including adult children, who often volunteer to care for their aged parents even at the cost of sacrificing their professional careers. The study proposes a formal care partner role for family members to help older adults navigate the digital health system. The observation not only advances the potential of catalyzing diversity as a “strength” for quality care but also identifies normative variations, which are seldom acknowledged in people-centered care policies. Within the intersectionalities of ethnicity, aging, and quality care principles, the intervention model provides a compelling argument for the segregation of data for “older ethnic adults”; it further reflects the need for digital determinants to distinguish between assumptions of typical “safety-net” traits of (minority) ethnic adults and their actual role as unique partners in strengthening the scope, scale, and equity of the health system and digital health.

Despite a noticeable increase in the acknowledgment of ethnically diverse data for the effective diagnosis of disease profiles and trajectories, ethnic patients remain the “subject” of studies rather than their architects. In the general discourse of health disciplines, data on ethnic minority groups are routinely aggregated with *vulnerabilities* and *marginalization concerns.* Yet, research about the impact of equity in the transformation of health systems, and more critically understanding the role of intersector approaches to challenges and opportunities of equity, would allow digital health to become more inclusive and sustainable.

Quoting science philosopher Thomas Kuhn, Meskó and colleagues [8] describe inequities in health as *anomalies* within the traditional paradigm of health that cannot be addressed without a shift in the structure of the system. This misalignment of the framework and the vision is described as a *paradox.* The conventional insular norms of health care systems act as barriers to equity, disregarding pluralism as deviation. The design of virtual care is susceptible to translating systemic inequities that may be embedded in existing models of health care. Crucial reform initiatives of cultural interventions [1] or designing methodical frameworks for equity analysis in digital health [9] are promising approaches for improving the equality aspects of health care, even if within the traditional systemic norms. To the extent the initiatives align with the culture-specific norms of the conventional system, targeted reform initiatives present good alternatives for improving the efficacy of care. Yet, in the absence of intentional transformative approaches, cultural variations in normative principles of health equity and digital health would continue to be interpreted as part of generic data, *measured by metrics of seemingly homogeneous vulnerabilities.* The unmet care needs and, more importantly, the cultural values that frame these needs—often acknowledged as proverbial cracks and gaps in quality of care—remain unrecognized as upstream factors and are seldom identified as a rationale for transformation.

## Equity Issues in Virtual Care

The concept of digital health equity is complex and multidimensional. It integrates a comprehensive consideration of individual contexts, the social determinants of health, and the enabling environment [10,11].

In the case of virtual care, the *complexity* increases with the introduction of digital determinants into the equation. The synoptic review of various models for health equity by Shaw et al [5] provides a glimpse of this complexity in their discussion of the levels of the digital divide, where individuals in the final level, who have access to technology and possess digital literacy, in addition to having competencies in digital navigation, are still not always able to achieve quality outcomes.

As the syndemic approach illustrates, epidemiologic assumptions about health equity generally address clinical-level efficiencies in the care quality of vulnerable ethnic minority groups, and seldom introduce the social lens into the equation. Inequities of digital health originate when the conventional approach gets instinctively coded into algorithms of digital technology, despite its innovative performance in various fields of medicine [11].

As long as the foundation of virtual care—the traditional health care system—remains unaware of its systemic cultural bias, innovative digital technologies and tools will mirror inequities. Virtual care represents a unique medium of care service delivery and, as such, it can effectively design technical solutions for access issues for all Canadians, including ethnic older adults and minority language–speaking patients, through the creation of appropriate user-friendly platforms for overcoming barriers to participation in digital health. Yet, substantive accommodation of ethnicity in contemporary discourse on cultural equity in health and digital health designs requires a shift in paradigm in the content of health care policy principles and its strategic priorities and action imperatives, which should resonate with the values of *all* patients and consumers *as aligned with the principles of pluralism*. Nothing less will help to achieve the purpose described by Pham et al [1] of catalyzing the transition from an exclusive service to an equitable standard.
